# Development, Optimization, and Validation of a Quantitative PCR Assay for *Borrelia burgdorferi* Detection in Tick, Wildlife, and Human Samples

**DOI:** 10.3390/pathogens13121034

**Published:** 2024-11-23

**Authors:** Julie Lewis, Vett K. Lloyd, Gilles A. Robichaud

**Affiliations:** 1 Department of Chemistry and Biochemistry, Université de Moncton, Moncton, NB E1A 3E9, Canada; 2 Department of Biology, Mount Allison University, Sackville, NB E4L 1G7, Canada; 3 Atlantic Cancer Research Institute, Moncton, NB E1C 8X3, Canada

**Keywords:** *Borrelia burgdorferi*, Lyme disease, ticks, wildlife, *Borrelia* culture, qPCR, real-time PCR

## Abstract

Tick-borne pathogens are growing in importance for human and veterinary research worldwide. We developed, optimized, and validated a reliable quantitative PCR (qPCR; real-time PCR) assay to assess Borrelia burgdorferi infection by targeting two B. burgdorferi genes, *ospA* and *flaB*. When assessing previously tested tick samples, its performance surpassed the nested PCR in efficiency, sensitivity, and specificity. Since the detection of *Borrelia* is more difficult in mammalian samples, the qPCR assay was also assessed using wildlife tissues. For wildlife samples, the sensitivity and specificity of *ospA* primers, with the incorporation of a pre-amplification step, was equivalent or superior to the nested PCR. For human samples, no primer set was successful with human tissue without culture, but we detected Borrelia with *ospA* and *flaB* primers in 50% of the Lyme culture samples, corresponding to 60% of the participants with a Lyme disease diagnosis or suspicion. The specificity of amplification was confirmed by Sanger sequencing. The healthy participant culture samples were negative. This PCR-based direct detection assay performs well for the detection of *Borrelia* in different biological samples. Advancements in detection methods lead to a better surveillance of *Borrelia* in vectors and hosts, and, ultimately, enhance human and animal health.

## 1. Introduction

Ticks are hematophagous ectoparasites that can transmit a variety of pathogenic and non-pathogenic microorganisms, including bacteria, viruses, protozoa, fungi, and nematodes, to human and animal hosts [1,2,3,4,5]. As the climate changes, tick populations are establishing and expanding, which impacts the incidence and locations of tick-borne diseases [6,7,8,9,10]. Numerous studies document the growing burden of tick-borne diseases and predict that it will continue to increase in many countries around the world [11,12,13,14,15,16,17,18]. These reasons drive the need for reliable tools for the direct detection of tick-borne pathogens, in both tick vectors and hosts.

Prominent among tick-borne pathogens is the *Borrelia burgdorferi* sensu lato complex, which encompasses an increasing number of closely related *Borrelia* species [19,20]. The most extensively studied and globally distributed Lyme-disease-causing *Borrelia* species is *B. burgdorferi* sensu stricto or *B. burgdorferi.* This bacterium is vectored by *Ixodes* tick species. As ticks feed, they can acquire infections from infected wildlife hosts and then transfer infections to uninfected hosts or uninfected co-feeding ticks [21,22,23]. When introduced into accidental hosts such as humans, Lyme disease, also known as Lyme borreliosis, results. This disease is a debilitating multisystemic disease, with potentially fatal outcomes [24,25,26,27,28,29]. Bacterial colonization begins with local infection in the extracellular matrix of the host skin, which acts as a protective niche for the bacterial replication [30,31]. Meanwhile, the underlying innate immune responses may lead to non-specific flu-like symptoms and the appearance of erythema migrans (EM) or other rashes [31,32,33,34,35,36,37,38]. Days to weeks following infection, the dissemination of *B. burgdorferi* progresses through the blood and the lymphatic circulatory systems to reach secondary sites of colonization. In immune-privileged tissues such as joints or central nervous tissues, the bacteria can further multiply [31,39,40]. At this point, symptoms may include secondary EM rashes, fatigue to an extent that impedes life activities, cognitive impairment, and neurological or arthritic afflictions, as well as other manifestations such as ocular and cardiac problems [34,41,42]. In addition to proliferation in immune-privileged sites, *B. burgdorferi* employs numerous strategies to evade and suppress the host’s innate and adaptative immune system [30,31,43,44,45,46,47,48,49]. Months to years post-infection, damage to the tissues may occur and late or chronic Lyme disease ensues [44,47,50,51,52]. Even among those treated promptly and fully by the accepted treatment modalities, approximately 14% have persistent symptoms [52] and the proportion with persistent symptoms increases with a delay in diagnosis [52]. Thus, the early detection of infection and prompt treatment have the best chance of leading to a full recovery [47,52,53,54,55,56,57,58,59,60,61].

The detection of a *Borrelia* infection in human samples is challenging for many reasons. In the clinical setting, the detection of *Borrelia* infection is indirect as it relies on the serological detection of anti-*Borrelia* antibodies and this system does not reliably detect the *Borrelia* infection in certain cases [49,53,54,61,62,63,64,65,66,67,68,69,70,71]. To address the deficiencies of indirect testing, direct pathogen detection methods such as nested PCR (nPCR) and quantitative PCR (qPCR; real-time PCR), with or without enrichment by culture, have been extensively used to detect *Borrelia* DNA in research [72,73,74,75,76]. While many PCR assays for *Borrelia* have been developed, not all are thoroughly optimized and validated. Those that have been validated tend to be validated only for one type of biological sample [77,78,79,80,81,82,83,84,85,86,87,88,89,90,91,92,93,94,95,96,97,98]. In this article, we describe a validated, optimized, accessible, and reliable qPCR assay targeting *Borrelia* species using a plasmid gene (*ospA*, *Outer surface protein A*) and a chromosomal gene (*flaB*, *flagellin B*). After the development, optimization, and validation of the protocol using pure *Borrelia burgdorferi* cultures alone or spiked into human DNA as a positive control, we used this assay to test different biological sample types, including ticks, tissues from reservoir wildlife species, and culture-amplified samples of humans diagnosed with Lyme disease. The sequencing of the resulting amplicons substantiated the primers’ specificity.

## 2. Materials and Methods

### 2.1. Sample Collection and Preparation

For the positive control, we used *Borrelia* DNA (*Borrelia burgdorferi* strain B31, ATCC #35210, the American Type Culture Collection, Manassas, VA, USA) spiked into human DNA to replicate the nature of clinical samples as much as possible. We extracted the human DNA from human embryonic kidney cells (HEK-293, ATCC # CRL-1573), lymphocyte B cells from a patient with lymphoma (Raji, ATCC # CCL-86), a cell line from peripheral blood of a patient with chronic lymphoid leukemia (Mec-1, DSMZ # ACC-497, the Leibniz Institute’s German Collection of Microorganisms and Cell Culture, Braunschweig, NI, DEU), and a cell line derived from clear cell adenocarcinoma from human kidney (786-0, ATCC # CRL-1932). The maintenance of the *Borrelia* pure culture was carried out using standard procedures [99] and as instructed by the bacteria provider (ATCC). Human cell culture was carried out as instructed by the providers (ATCC and DSMZ). Cells were counted using a hemacytometer and trypan blue (Gibco, Thermo Fisher Scientific, Waltham, MA, USA) to assess cell number for DNA extraction. Phosphate-buffered saline (PBS) 1× was added to the cell pellet, obtained by centrifugation at 2000× *g* for 10 min, before DNA extraction was performed.

Tick samples came from the Lloyd Lab Tick Bank at Mount Allison University (Sackville, NB, Canada). Ticks were obtained by passive surveillance and donated by the public and veterinary clinics in 2020. Table 1 provides information on the origin of the ticks, as reported by tick donors, and information on tick species, life stage, sex, engorgement, and *Borrelia burgdorferi* presence or absence. *B. burgdorferi* infection was determined by nested-PCR-targeting the 16S-23S rRNA intergenic sequence region, as described by Lewis et al. (2021) [100]. Ticks (n = 30) were selected to include 10 ticks from each of the three categories: negative, positive, and ambiguous for the presence of *B. burgdorferi* DNA. Ticks from the ambiguous category are ticks for which the gel result showed amplicons of unexpected size or weak amplicons of the correct size.

Wildlife samples were collected in New Brunswick (Canada) as described previously [101]. Briefly, a non-targeted approach was used to collect medium (raccoons, porcupines, etc.) and small (rodents, shrews, etc.) animals to maximize the number and diversity of species collected. Animals were identified morphologically. Small animals were frozen upon collection until tissues were dissected whereas dissection of larger animals occurred at the site of recovery. DNA was extracted from the dissected tissues as described by Zinck and Lloyd (2022) [101], and tested for *B. burgdorferi* by nested PCR [101], and residual DNA was stored at −20 °C. The previously extracted and frozen DNA from 21 of those samples was used in this study. Table 2 provides information on the wildlife samples.

Human tissue samples (kidney, liver, pancreas, tooth, pericardium, endocardium, cerebral cortex, aorta, tricuspid valve, and pulmonary vein) were obtained from the Lyme disease biobank (Mount Allison Research Ethics Board, approbation # 2016-042/101796) from three individuals diagnosed with Lyme disease at the time the samples were removed.

The culture samples, derived from individuals with a diagnosis or suspicion of Lyme disease, were acquired from clinical collaborators, Marianne Middelveen (Atkins Veterinary Services, Calgary, AB, Canada) and Dr. Raphael Stricker (Union Square Medical Associates, San Francisco, CA, USA) in 2016 and 2017 (research ethics approval # 1148461). Table 3 contains information on the samples collected and the demographic data provided by study participants. All of these participants were from Alberta (Canada), except for participant L12, who resided in Ontario (Canada). Medical and diagnostic history were provided by the participants. All participants had been experiencing long-term (months to years) chronic illness, which would exclude the possibility of acute Lyme disease. As is common for “real-world” samples, access to primary or specialist healthcare was variable so not all participants had access to conventional two-tiered serological testing or private serological testing using alternate diagnostic approaches. Some participants were under the care of relevant specialists (5 different physicians) but most did not have access to specialist care.

All the samples and cultures tested in this study were used exclusively for this study. Participants L2, L3, L5, L6, L7, L12, and L20 had previously donated samples to be cultured with findings described by Middelveen et al. (2014) (using different participant codes) [102]. Participants L2, L3, L6, L7, and L20 also provided samples for analysis described (using different participant codes) in Middelveen et al. (2018) [103]. We additionally obtained 6 negative biological control samples, which consisted of BSK-H cultures derived from 3 genital and 3 blood samples from 6 individuals with no Lyme disease diagnosis or suspicion. These samples were collected by Dr. John Haggblad (Associate Clinic, Calgary, AB, Canada) and cultured by Middelveen (ethical approval # HREBA.CHC-15-0029). Samples were cultured by Middelveen using the Barbour–Stonner–Kelly (BSK-H) medium (Dalynn Biologicals, Calgary, AB, Canada) with 6% rabbit serum (Sigma-Aldrich, # B8291, St. Louis and Burlington, MA, USA), a medium optimized for the growth of *Borrelia* [96,97], to which three antibiotics (Sigma-Aldrich, St. Louis and Burlington, MA, USA) were added: amphotericin B (2.5 μg/mL), rifampicin (50 μg/mL), and phosphomycin (20 μg/mL). Cultures were incubated between 34 to 35 °C for 4 weeks and sent to Mount Allison University (MTA), where they were stored at 4 °C for a few to approximately 90 days, until DNA extraction was performed. We obtained 24 BSK-H cultures derived from different samples from 15 Lyme disease patients. The different samples used to make the Lyme BSK-H cultures consisted of a skin sample (n = 1), urine samples (n = 3), genital samples (semen or vaginal swab; n = 15), periodontal samples (n = 2), and synovial fluid from the knee (n = 2) and the ankle (n = 1). Some of these samples included biological replicates, obtained from the same sample type of the same participant but collected at different times.

### 2.2. DNA Extraction

We started the optimization and validation process by assessing different DNA extraction kits. Five different DNA extraction methods were used to extract DNA from *B. burgdorferi* pure cultures: AquaGenomics (MultiTarget Pharmaceuticals LLC, Salt Lake City, UT, USA), DNeasy Blood & Tissue kits (Qiagen, Germantown, MD, USA), Purelink Genomic DNA Mini Kit (Thermo Fisher Scientific, Waltham, MA, USA), Monarch^®^ Genomic DNA purification Kit (New England BioLabs, Ipswich, MA, USA), or Extracta DNA Prep for tissue (Quantabio, Beverly, MA, USA). The human genomic DNA was extracted using the PureLink Genomic DNA kit (Thermo Fisher Scientific, Waltham, MA, USA) and the QIamp DNA mini kit (Qiagen, Hilden, NRW, DEU). The DNA extraction from the *B. burgdorferi* pure cultures and the human cell lines was performed as described by each of the manufacturers’ protocols. DNA concentrations of both the *Borrelia* and human DNA was determined using a nanodrop ND-1000 spectrophotometer (Thermo Fisher Scientific, Waltham, MA, USA), with the respective DNA resuspension solutions as a blank. To prepare the spiked positive controls, different concentrations of *Borrelia* DNA (depending on the experiment conducted, as described in Section 2.4) was mixed with 100 ng of human DNA and samples were stored at −20 °C. For the assessment of the optimized qPCR protocol with different biological sample types, different DNA extraction protocols were not exhaustively tested as sample availability was limited. The choice of DNA extraction protocol was based on expediency or prior choice for archived DNA samples.

For DNA extraction of BSK-H cultures derived from Lyme samples, the AquaPlasmid reagent (MultiTarget Pharmaceuticals LLC, Salt Lake City, UT, USA) was used, following the manufacturer’s protocol. Briefly, samples were centrifuged at 10,000× *g* for 10 min in a benchtop microcentrifuge (Spectrafuge^TM^ 24D Microcentrifuge, Labnet International, Cary, NC, USA) to pellet bacteria, which was resuspended in 100 to 200 μL of PBS 1× and put at −80 °C overnight to aid in lyzing the bacteria. Samples were then centrifuged as before and resuspended in 200 μL of AquaPlasmid solution, vortexed, and incubated at −20 °C for 5 min, and the cellular debris was pelleted by centrifugation at 14,000× *g* for 5 min. The supernatant was precipitated with 0.5 volume isopropanol, centrifuged at 14,000× *g* for 5 min, washed with ethanol (70%), air-dried, and resuspended in 50 μL of a Tris 1 mM pH 7 solution. DNA samples were stored at −20 °C until qPCR testing.

Tick DNA extraction was performed using the AquaGenomic reagent (MultiTarget Pharmaceuticals LLC, Salt Lake City, UT, USA) according to the manufacturer’s protocol. Briefly, ticks were homogenized in 50 to 200 μL of AquaGenomic solution, depending on the size and engorgement status of the tick, by using an Eppendorf pestel (Diamed Lab Supplies Inc., Mississauga, ON, Canada). The samples were incubated at 60 °C for 45 min to inactivate nucleases. The samples were then centrifuged at 16,300× *g* in a benchtop microcentrifuge (Spectrafuge^TM^ 24D Microcentrifuge, Labnet International, Cary, NC, USA) and the supernatant precipitated with an equal volume of isopropanol. After pelleting the DNA by centrifugation, the pellet was washed with 50 μL of ethanol (70%), air-dried at room temperature, and resuspended in 50 μL of 1 mM Tris pH7 by incubating at 60 °C for an hour. DNA samples were stored at −20 °C until qPCR testing.

For the wildlife samples and human tissue samples, DNA was extracted as described previously [101] using the AquaGenomic reagent (MultiTarget Pharmaceuticals LLC, Salt Lake City, UT, USA), according to the manufacturer’s protocol as described for ticks above, with the exception that tissue samples were pre-digested in proteinase K (5 μg) in 100 μL of AquaGenomic solution for 90 min at 55 °C followed by a 10 min deactivation step at 95 °C before proceeding with the standard DNA extraction protocol. To confirm the DNA integrity of these archived samples (wildlife, human tissue, and tick samples), amplification of the *mitochondrial Cytochrome C Oxidate subunit 1* (*CO1*) was used (Table 4). The PCR reaction mix consisted of the same reagents and volumes as described for the two rounds of conventional PCR with outer primers and the semi-nested PCR (Section 2.5). The *CO1* PCR program was as follows: an initial denaturation of 94 °C for 5 min; 5 cycles of 94 °C for 30 s, 45 °C for 30 s, and 72 °C for 1 min; and 35 cycles of 94 °C for 30 s, 51 °C for 30 s, and 72 °C for 1 min.

### 2.3. Primer Design

Primers targeting a fragment of two *Borrelia* genes (*ospA* or *outer surface protein* and *flaB* or *flagellin B*) were designed for the qPCR assay. Ten primer pairs were generated for each gene using the software Primer3Plus (version 3.3.0), based on the criteria recommended by Thornton and Basu [104]. To detect the maximum of *Borrelia* species possible, several sequences from different *Borrelia* species (GenBank # AE000790.2:9457-10278, CP002761.1:9504-10325, CP003202.1:c45988-45170, and AB253532.1 for the *ospA* primer pair; GenBank # CP014808.1:778274-779278, NC_022048.1:c148659-147649, NZ_CP007564.1:753937-754947, CP000013.1:c148255-147245, CP021872.1:147918-148925, CP002746.1:147244-148254, and NZ_CP009212.1:147324-148334 for the *flaB* primer pair) were aligned in the MUSCLE (Multiple Sequence Comparison by Log-expectation) software version 5 on the EMBL-EBI (European Molecular Biology Laboratory of the European Bioinformatics Institute, Hinxton, CAM, UK) website. Of the 10 primer pairs, the one with the most conserved regionmins between species were selected for each of the target genes. The selected *ospA* and *flaB* primer pairs were inspected for primer dimers and secondary PCR products using the following resources: Oligocalc: Oligonucleotide Properties Calculator version 3.27, and Mfold web server for nucleic acid folding and hybridization prediction version 3.0, as well as AmplifyX version 2.1.1 to simulate the PCR in silico. Primers with a %CG between 40 and 60 were then chosen outside the previous primer pairs and inspected with the same resources as described above. Primer BLAST (National Center for Biotechnology Information (NCBI), Bethesda, MD, USA) version 2.10.0 was used to confirm the specificity of the primers. *OspA* and *flaB* primers were manufactured by Integrated DNA Technology (IDT, Coralville, IA, USA). The *ospC* and *CO1* [105] primers were taken from the literature and manufactured by Sigma-Aldrich (St. Louis and Burlington, MA, USA). Table 4 shows the primer sequences, the hybridization temperature, and the amplicon length for all the primer pairs used in this study.

**Table 4:** PCR primers and amplification conditions.

Target	Type of PCR	Primer Name	Sequence (5′ → 3′)	Hybridization Temperature (°C)	Amplicon Length (bp)	Source of the Primers
*Borrelia* species	qPCR ^1^	*ospA* forward	GAACCAGACTTGAATACACAGGA	61	98	Designed for this study
		*ospA* reverse	TTCAGCAGTTAGAGTTCCTTCA			
		*flaB* forward	CAATCAGGTAACGGCACATATTC	61	80	
		*flaB* reverse	CTATTAATTTCGTCTGTAAGTTGCTC			
	Two rounds of conventional PCR and outer round of semi-nested PCR ^2^	outer *ospA* forward	CAAGAGCAGACGGAACC	52.5	190	
		outer *ospA* reverse	AGTTCAACTGAAACTTCCC			
		outer *flaB* forward	GTAAGAATGAAGGAATTGGC	52.5	204	
		outer *flaB* reverse	AGGCTGCATTCCAAGCTC			
	Inner round of semi-nested PCR ^3^	*ospA* forward	GAACCAGACTTGAATACACAGGA	53.5	178	
		outer *ospA* reverse	AGTTCAACTGAAACTTCCC			
		*flaB* forward	CAATCAGGTAACGGCACATATTC	61	180	
		outer *flaB* reverse	AGGCTGCATTCCAAGCTC			
	Nested PCR ^4^	outer *ospC* forward	GTAATAATTCAGGGAAAGATGG	51	654	
		outer *ospC* reverse	CAGCACCTTTAGTTTTAGTACC			
		inner *ospC* forward	CTAATGCGGTTTTACTTGCTG	56	331	
		inner *ospC* reverse	GTTTTTAAAATGGCTTCTTTTGC			
Diverse animals	Conventional PCR ^5^	*CO1* forward	GGTCAACAAATCATAAAGATATTGG	45 (5 cycles) 51 (35 cycles)	710	Folmer et al. (1994) [105]
		*CO1* reverse	TAAACTTCAGGGTGACCAAAAAATCA			

^1^ The qPCR program had an initial denaturation at 95 °C for 3 min, followed by 40 cycles of denaturation at 95 °C for 15 s, hybridization at 61 °C for 30 s, and elongation at 72 °C for 30 s, as described in Section 2.4. ^2^ The PCR program for both rounds of conventional PCR and for the outer round of the semi-nested PCR was as follows: 95 °C for 10 min; 20 cycles at 95 °C for 1 min, 52.5 °C for 30 s, and 72 °C for 30 s; and 72 °C for 5 min. ^3^ The PCR program for the inner round of the semi-nested PCR was the same as the outer round but with an annealing temperature of 53.5 °C and the number of cycles increased to 40, as described in Section 2.5. ^4^ The *ospC* program for the outer round was as follows: an initial denaturation of 94 °C for 5 min; 35 cycles of 94 °C for 45 s, 51 °C for 45 s, and 72 °C for 45 s; and a final extension of 72 °C for 5 min. The program for the *ospC* inner round was the same as the outer round except for the annealing temperature which was 56 °C instead of 51 °C, as described in Section 2.5. ^5^ The *CO1* PCR program was as follows: an initial denaturation of 94 °C for 5 min; 5 cycles of 94 °C for 30 s, 45 °C for 30 s, and 72 °C for 1 min; and 35 cycles of 94 °C for 30 s, 51 °C for 30 s, and 72 °C for 1 min, as described in Section 2.2.

### 2.4. Validation and Optimization of the qPCR Assay

Before testing tick and human samples, the qPCR assay was validated and optimized. The first step was to test different qPCR master mixes. Quantabio’s PerfeCta 2× SYBR Green SuperMix (VWR 101414, Radnor, PA, USA), Quantabio’s PerfeCta 2× FastMix II (VWR 97065, Radnor, PA, USA), and Montreal Biotech MBI EvOlution 5× EvaGreen qPCR mix (Montreal Biotech MBI-E1000, Dorval, QC, Canada) were tested to determine which one was the most efficient for our samples. This was performed with technical triplicates, for the *ospA* and *flaB* primers, at different concentrations of *Borrelia* (10 ng, 1 ng, and 0.1 ng) in 100 ng of human DNA. We also included samples of 100 ng of only *Borrelia* DNA and 100 ng of only human DNA. To further evaluate the detection limit, a sensitivity test was performed, again with technical triplicates and the same primers but using samples that included an even lower concentration, 0.001 ng of *Borrelia* DNA in 100 ng of human DNA. Secondly, a hybridization temperature gradient, between 58 °C and 63 °C, was performed to select the optimal hybridization temperature, one with good specificity without much compromise of sensitivity, again using technical triplicates for *ospA* and *flaB* primers and with the same templates as described above. Specificity of primer pairs was assessed through the inspection of the melt curve peaks for secondary peaks and primer sensitivity was assessed by looking at the fluorescence (*Y* axis) of the melt curve peaks. A PCR amplification efficiency test was then performed for the *Borrelia* primers (*ospA* and *flaB*) by using a serial dilution (1:10) of the *Borrelia* DNA sample, starting with a quantity of 100 ng.

For the first of the validation and optimization steps, the performance of the different previously mentioned qPCR master mixes was compared and, for the rest of the steps, the SYBR Green Super mix (the commercial qPCR master mix that produced the best amplification—see Section 3.1) was used. The PCR reaction mix also consisted of 450 nM of forward primer, 450 nM of reverse primer, and 2 μL of DNA template in a final volume of 10 μL. For each of the steps, a negative control (no-template control) was carried out in triplicate for each primer pair tested. The PCR program had an initial denaturation at 95 °C for 3 min, followed by 40 cycles of denaturation at 95 °C for 15 s, hybridization at 61 °C for 30 s, and elongation at 72 °C for 30 s. The melt curve was carried out between 65 °C and 95 °C with an increase of 0.5 °C every 5 s. A CFX96 Connect qPCR Detection System thermocycler (Bio-Rad, Hercules, CA, USA) was used, and data were obtained using the software CFX Maestro 1.1, version 4.1.2433.1219.

### 2.5. Testing of Tick Samples, Wildlife Tissues, Human Tissues, and BSK-H Cultures Derived from Human Samples

Ticks from the positive, negative, and ambiguous categories were tested with the optimized qPCR protocol, with technical triplicates, using *Borrelia* primers (*ospA* and *flaB*). BSK-H cultures derived from samples of individuals with a Lyme disease diagnosis or suspicion of Lyme disease, as well as negative controls (BSK-H cultures derived from those with no diagnosis or suspicion of Lyme disease), were also tested by qPCR with technical triplicates, using *Borrelia* primers (*ospA* and *flaB*). Testing of these samples was carried out simultaneously with a negative control (no-template control) and a positive control (100 ng of *Borrelia* DNA). For the testing of the biological samples, the PCR reaction mix, the PCR program, the thermocycler, and the software used to obtain the data were the same as described for the validation and optimization steps (Section 2.4). The *ospC*-nested PCR testing was performed on BSK-H culture samples using the MultiGene OptiMax thermal cycler (Labnet International, Cary, NC, USA). The PCR reaction mix was carried out in a final volume of 25 µL, which included 12.5 µL of GoTaq Green 2× Master mix (Promega, Madison, WI, USA), 8.5 µL of nuclease-free water, 1 µL of forward primers (10 µM), 1 µL of reverse primers (10 µM) (primers are listed in Table 4), and 2 µL of DNA (or nuclease-free water for the no-template control) for the outer round and PCR product of the outer round for the inner round. The program for the outer round was as follows: an initial denaturation of 94 °C for 5 min; 35 cycles of 94 °C for 45 s, 51 °C for 45 s, and 72 °C for 45 s; and a final extension of 72 °C for 5 min. The program for the inner round was the same as the outer round, except for the annealing temperature, which was 56 °C instead of 51 °C.

For wildlife and human tissue samples, DNA extracted directly from tissues was tested by qPCR in duplicate with and without a round of pre-amplification for the *Borrelia ospA* and *flaB* genes using the iCycler iQ qPCR Detection System (Bio-Rad, Hercules, CA, USA) and iCycler software version 3.0.6070. The PCR reaction mix and PCR program are as described for the validation and optimization steps (Section 2.4). These samples were also tested with two rounds of conventional PCR using outer *ospA* and *flaB* primers and with a semi-nested PCR using outer forward and reverse primers for the first round or forward primer and outer reverse primer for the second round, as listed in Table 4 (outer *ospA* or *flaB* reverse and *ospA* or *flaB* forward) using the MyCycler Thermal Cycler (Bio-Rad, Hercules, CA, USA). The PCR reaction mix used the same reagents and volumes as for *ospC.* The PCR program for both rounds of conventional PCR and for the outer round of the semi-nested PCR was as follows: 95 °C for 10 min; 20 cycles at 95 °C for 1 min, 52.5 °C for 30 s, and 72 °C for 30 s; and 72 °C for 5 min. The PCR program for the inner round of the semi-nested PCR was the same as the outer round but with an annealing temperature of 53.5 °C and the number of cycles increased to 40.

### 2.6. DNA Sequencing

To confirm the specificity of amplification, some of the amplicons were Sanger-sequenced. The short qPCR amplicons were prepared for sequencing by cloning, whereas longer amplicons (two round of conventional PCR, semi-nested PCR, and nested PCR) were directly sequenced. Of the biological samples that amplified a short qPCR product, 8 samples consisting of *ospA* and *flaB* amplicons obtained from tick samples, Lyme BSK-H cultures, and the positive control (DNA extracted from the *B. burgdorferi* pure culture) were prepared for Sanger sequencing. This was carried out by cloning and purifying plasmid DNA according to the manufacturer’s protocol of the kits and materials. Briefly, the PCR product migration was carried out in a 2% agarose gel for approximately 1 h at 120 volts; bands corresponding to the amplicon’s sizes were removed from the gel, from which DNA was extracted using the gel extraction kit (Qiagen, Germantown, MD, USA). The PCR products were ligated in the cloning vector pGEM by using the pGEM-T Easy vector systems (Promega, Madison, WI, USA) and plasmid DNA was transformed into competent cells using *Escherichia coli* Mix and Go JM109 (Zymo Research, Irvine, CA, USA). The colonies were then screened with regular PCR using the commercial PCR mix DreamTaq PCR Master Mix (2×) (Thermo Fisher Scientific, Waltham, MA, USA) with the same *ospA* and *flaB* primers as described for qPCR in Section 2.3. The selected colonies were then cultured in 2 mL of liquid LB culture medium at 37 °C in a shaking incubator. Plasmid DNA was extracted using the ZymoPURE Plasmid Miniprep kit (Zymo Research, Irvine, CA, USA) and sent to the TCAG DNA sequencing facility (Toronto, ON, Canada) for Sanger sequencing of DNA strands was done in both directions (sense and antisense). For the sequencing of the longer amplicons, 11 *ospA* amplicons and two *flaB* amplicons from wildlife samples were obtained using two rounds of conventional PCR, five *ospA* and one *flaB* amplicons from the wildlife samples were obtained using semi-nested PCR, two *ospA* amplicons and two *flaB* amplicons from ticks of the positive category were obtained by two rounds of conventional PCR, and seven *ospC* amplicons from BSK-H cultures were obtained with nested PCR. Longer amplicons were sent to Centre d’expertise et de services Génome Québec (Montréal, QC, Canada). The sequences were then manually compared using the FinchTV software version 1.4.0, and a nBLAST analysis, optimized for highly similar sequences (megablast) (NCBI, the National Center for Biotechnology Information, Bethesda, MD, USA), was performed on the consensus sequences.

## 3. Results

### 3.1. Validation and Optimization Results of the Borrelia qPCR Assay

Before testing the tick, wildlife, and human samples for the *Borrelia* species, the qPCR assay was validated and optimized. First, the DNA extraction methods (described in Section 2.2) for the *B. burgdorferi B31* pure cultures of the positive control were tested to determine which one had the best DNA yield, and the Extracta DNA prep for tissue (Quantabio) was found to perform best (see Table 5).

**Figure 1:**
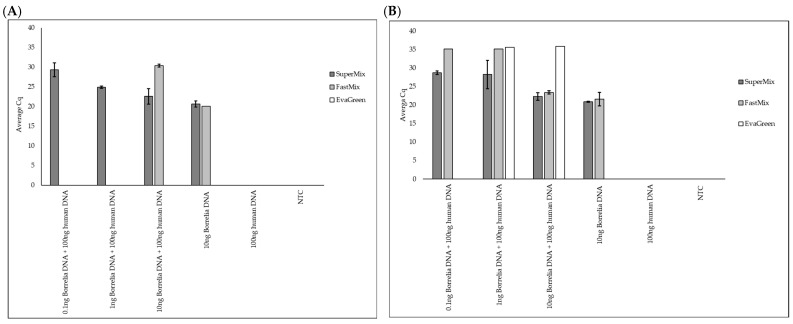
Quantitative PCR assay results for the optimization of the commercial qPCR master mix test. The SYBR Green SuperMix is represented in dark gray, the FastMix is in light gray, and the EvaGreen Mix is in white, for the *ospA* (**A**) and *flaB* (**B**) primers. The average Cq values were calculated from the technical triplicate Cq values and the error bars were calculated using the standard deviation of the technical triplicate Cq values. The NTC sample is a no-template control.

Figure 1 shows the comparison of the performance of the three different commercial qPCR mixes tested (SYBR Green SuperMix, FastMix, and EvaGreen qPCR mix) with our *Borrelia* primers, using different concentration of *Borrelia* DNA spiked in 100 ng of human DNA. The SuperMix (dark gray, Figure 1) was the most sensitive qPCR mix, with detection between 20 and 30 cycles, lower than the other mixes, for all the concentrations tested, for both targeted genes (*ospA* in Figure 1A and *flaB* in Figure 1B). For the FastMix (light gray, Figure 1), *Borrelia* samples amplified a product from the most concentrated *Borrelia* DNA samples (i.e., 10 ng of *Borrelia* DNA in 100 ng of human DNA, approximately equivalent to 800 *Borrelia* genomes, constituting 8000–16,000 targets depending on the cell ploidy, in 60,000 human genomes) at 30 cycles of amplification and 10 ng of pure *Borrelia* DNA at 20 cycles with *ospA* (Figure 1A), whereas, for *flaB* (Figure 1B), *Borrelia* DNA was only detected with a low Cq value in the most concentrated *Borrelia* DNA samples (i.e., 10 ng of *Borrelia* DNA in 100 ng of human DNA and 10 ng of pure *Borrelia* DNA, both below 25 cycles, and a high Cq of 35 cycles was obtained for the two samples with the lowest *Borrelia* DNA concentration; 1 ng of *Borrelia* DNA in 100 ng of human DNA, or approximately 80 *Borrelia* genomes in 60,000 human genomes, and 0.1 ng *Borrelia* DNA in 100 ng human DNA, or approximately 8 *Borrelia* genomes in 60,000 human genomes). For the EvaGreen qPCR Mix (white, Figure 1), no amplification, except for the amplification of two samples with high Cq values (10 ng of *Borrelia* DNA in 100 ng of human DNA and 1 ng of *Borrelia* DNA in 100 ng of human DNA, both appearing at 35 cycles) was obtained for *flaB*.

**Figure 2:**
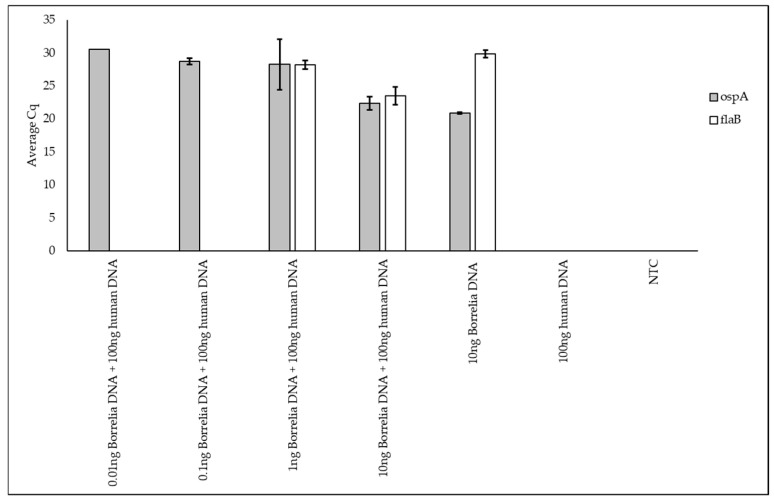
Detection limit of the qPCR *Borrelia* primers. The average Cq values, calculated from the technical triplicate Cq values, are represented in gray for *ospA* and in white for *flaB*. Error bars were calculated using the standard deviation of the technical triplicate Cq values. The NTC sample is a no-template control.

To determine the sensitivity of the primers, the next step was carried out by lowering the *Borrelia* DNA concentration (Figure 2). The *ospA* primers detected *Borrelia* DNA at even the lowest concentration of DNA tested (0.01 ng of *Borrelia* DNA in 100 ng human DNA at around 30 cycles), whereas the *flaB* primers detected nothing at this concentration or the next lowest *Borrelia* concentration (0.1 ng of *Borrelia* DNA in 100 ng human DNA). These results indicate that the limit of detection for *Borrelia* DNA is 0.01 ng or lower for the *ospA* primers and between 0.1 ng to 1 ng for *flaB*.

**Figure 3:**
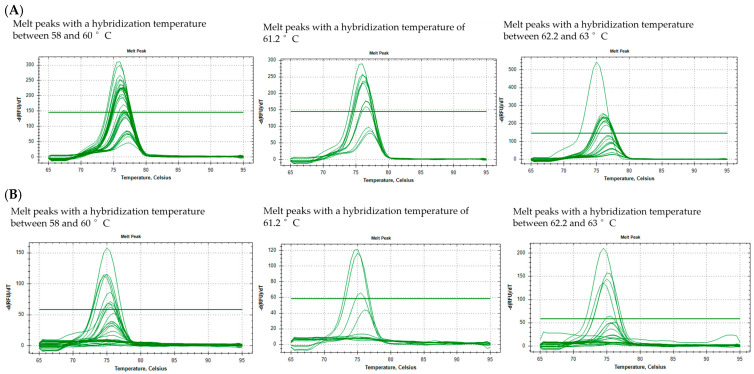
Optimization of hybridization temperature for the qPCR primers. The peak graphs show derivatives of the fluorescence as the melting temperature increases. The hybridization temperature gradient covered the following temperatures: 58, 59, 60, 61.2, 62.2, and 63 °C. The melt peaks for the two targeted *Borrelia* genes, *ospA* (**A**) and *flaB* (**B**), at different concentrations of *Borrelia* DNA (10, 1, and 0.1 ng) in 100 ng of human DNA and 100 ng of *Borrelia* DNA only are combined in these graphs. Hybridization temperatures were grouped (from 58 °C to 60 °C, 61.2 °C, and from 62.2 °C to 63 °C) in these graphs according to the changes observed in the peaks. More than one melt peak or the distortion of peaks indicate a non-specific amplification. The threshold line was placed by the CFX Maestro 1.1 software, version 4.1.2433.1219 based on default settings.

To further optimize the amplification sensitivity and specificity, the optimal hybridization temperature gradient was determined by varying the annealing temperatures between 58 °C and 63 °C and examining the melt curves to assess specificity (determined by a single peak) and sensitivity (the height of the peaks). These data are presented in Figure 3, for *ospA* (Figure 3A) and *flaB* (Figure 3B). With hybridization temperatures below 61.2 °C, a small secondary peak indicates that amplification loses specificity relative to other hybridization temperatures, for *ospA* and *flaB*. Thus, the annealing temperature chosen had to be 61.2 °C or higher. To choose between these temperatures (61.2, 62.2, or 63 °C), the sensitivity was assessed by looking at the fluorescence signal of graphs (*Y* axis) in Figure 3. At 61.2 °C, the signal of the peak rises to around 300 RFU (relative fluorescence units) for *ospA* and 120 for *flaB*. However, for 62.2 and 63 °C, the fluorescence signal starts to generally diminish, especially for *ospA*. Considering this, the hybridization temperature of 61 °C was chosen for *ospA* and *flaB* since it allows for a good specificity without compromising the sensitivity. A difference of fluorescence signal was noted between *ospA* and *flaB* (around 300 versus 120, respectively), which suggests that *ospA* primers are more sensitive than *flaB* primers.

**Figure 4:**
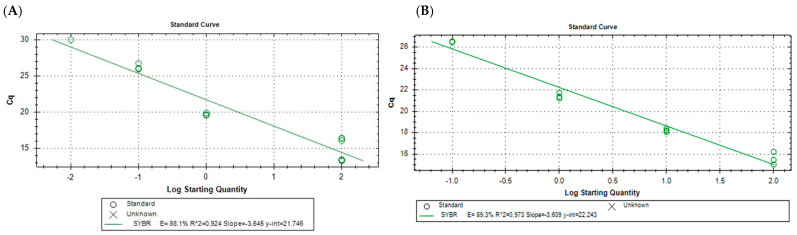
Efficacy test for the primers used in this study. The efficacy percentage (E) is shown along with the goodness-of-fit (R^2^) and the linear equation for the primers *ospA* (**A**) and *flaB* (**B**), as determined by the Maestro software 1.1, version 4.1.2433.1219. Standards are samples from a serial dilution (1:10) of *Borrelia* DNA starting with 100 ng of *Borrelia* DNA, in a volume of 2 µL, tested with technical triplicates.

The amplification efficiency for the qPCR primers *ospA* (Figure 4A) and *flaB* (Figure 4B) was calculated by the CFX Maestro software 1.1, version 4.1.2433.1219 with samples described in Section 2.4. The efficiencies of the primer pairs were similar, 88.1% for *ospA* and 89.3% for *flaB*. Comparing the slopes of the different primer pairs allowed us to determine that they have a similar amplification efficacy. The goodness-of-fit of the curve approaches 1, which ensures a good technique has been employed in manipulating samples.

### 3.2. PCR Testing on Tick, Wildlife, and Human Samples

#### 3.2.1. Quantitative PCR Testing on Tick Samples

**Figure 5:**
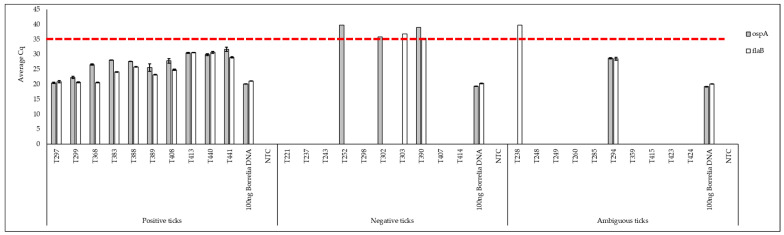
Quantitative PCR results of tick samples. Quantitative PCR for positive ticks, negative ticks, and ambiguous ticks. The classification of ticks as positive, negative, and ambiguous is based on nPCR testing [100]. The red line indicates the 35-cycle cut-off line; to be considered positive amplification, both genes must be non-zero values below this line. The average Cq results are shown in gray for *ospA* primers and in white for *flaB* primers. The samples starting with the letter “T” represent tick samples. The negative control consisted of a PCR reaction with molecular-grade water instead of DNA (NTC for no-template control) and the positive control was 100 ng of *Borrelia* DNA in a volume of 2 µL. The average Cq was calculated with the Cq values of technical triplicates and the error bars were calculated by using the standard deviation of the Cq values of the technical triplicates.

Figure 5 shows the results of the qPCR using our optimized primer sets on tick DNA. Amplicons were generated for all the ticks previously found to be *Borrelia*-positive with average Cqs between 20 and 30 cycles, for both genes (*ospA* and *flaB*). Ticks from the negative category did not produce any amplification or they had a signal of amplification of 35 cycles (average Cq) for both genes or for one of the two genes. For the ticks from the ambiguous category, amplification was obtained for only one tick and it occurred below 30 cycles for *ospA* and *flaB*. The positive control (100 ng of *Borrelia* DNA) amplified a product with an average Cq of 20 cycles for *ospA* and *flaB*. The negative controls (NTC) for *ospA* and *flaB* did not amplify, or amplification was at 35 cycles or more. Based on these results, the amplification of a PCR product with an average Cq below 35 cycles indicates a tick positive for the presence of *Borrelia* DNA.

#### 3.2.2. PCR Testing of Wildlife Samples

As the amplification of *Borrelia* is more challenging in mammals than ticks due to the overwhelming amounts of host DNA [101], we investigated both the basic qPCR amplification protocol and a pre-amplification step that might increase its sensitivity. Table 6 shows the testing outcome for the DNA extracted from 21 tissues samples from mammalian reservoir hosts, of which seven samples previously determined by nested PCR to have *Borrelia* and 14 without *Borrelia* [101]. Without any pre-amplification, none of the seven positive samples showed a positive result with qPCR for either the *ospA* and *flaB* primer sets; one of the negative samples (C3K16) showed amplification with the *ospA* and *flaB* primer sets and two produced non-specific amplification (non-*Borrelia* sequences) with the *flaB* primer set. With pre-amplification, three positive samples (R1K16, R1L16, and R6L16) and four samples previously categorized as negative (C3K16, C70K16, C94K16, and C6K17) showed amplification with the *ospA* primer set. Two other samples, one categorized as positive (C50K17) and the other as negative (C126K16), produced a poor-quality sequence using semi-nested PCR and a non-specific amplification using two rounds of conventional PCR, respectively. We also investigated two rounds of amplification with outer *ospA* and *flaB* primers, which gave only non-specific amplification for the samples previously categorized as negatives and a poor sequence quality for samples previously categorized as positive, although this did generate the amplification of *B. burgdorferi* in tick samples (see the *ospA* amplicon of T299 and *flaB* amplicons of T299 and T375 in Supplemental Tables S1 and S2), and semi-nested amplification with the primers listed in Table 4. The semi-nested PCR produced *Borrelia* amplicons for one sample categorized as positive (R1L16) and four samples categorized as negatives (C3K16, C70K16, C94K16, and C6K17), all identified as positive for *ospA* by pre-amplification followed by qPCR. The amplicons generated by the semi-nested PCR were large enough for Sanger sequencing without a preliminary cloning step; the *ospA* amplicons and the *flaB* amplicon were confirmed to detect *B. burgdorferi* (Supplementary Table S2). Thus, the *ospA* primer set is effective in detecting *B. burgdorferi* in wildlife tissues when adapted with a pre-amplification step [101]. Consistent with our findings during validation, the *flaB* primer set does not appear to be sufficiently sensitive or specific for unamplified mammalian tissue samples.

#### 3.2.3. Quantitative PCR Testing of Human Samples

We extended the use of our assay to test DNA from untreated *Borrelia* seropositive human tissues but were unable to identify amplicons with the *ospA* primer set (Supplemental Figure S1). Since the detection of *Borrelia* DNA in human samples is difficult, we also tested BSK-H cultures obtained from human samples.

**Figure 6:**
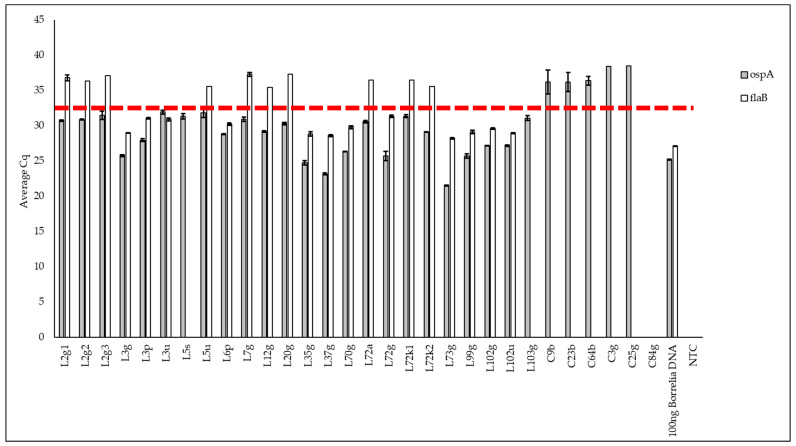
Quantitative PCR results of Lyme BSK-H cultures. Quantitative PCR results for the testing of BSK-H culture derived from Lyme and control samples. These samples were tested for two *Borrelia* genes (*ospA* in gray and *flab* in white). The red line indicates the 35-cycle cut-off line; to be considered positive amplification, both genes must be non-zero values below this line. The samples starting with the letter “L” are Lyme samples and samples starting with the letter “C” are BSK-H culture controls. The sample ending with the letter “s” is the skin sample, “g” is for genital samples, “u” is for urine samples, “p” is for periodontal samples, “a” is for the ankle synovial fluid sample, and “k” is for knee synovial fluid samples. The numbers at the end of the sample’s names (1, 2, or 3) represent the biological replicates. Negative controls consisted in molecular-grade water (NTC for no-template control) and the BSK-H cultures derived from control samples, provided by study participants that were not diagnosed with Lyme disease. The positive control was 100 ng of *Borrelia* DNA in a volume of 2 µL. The average Cq was calculated from the Cq values of the technical triplicates, and the error bar was calculated by doing the standard deviation of the technical triplicates.

Culture is a traditional means of amplifying *Borrelia* in a sample to enhance detection [99]. Figure 6 shows the result of qPCR on DNA of BSK-H cultures derived from samples from people with a diagnosis or suspicion of Lyme disease (Figure 6, “L” samples) and samples from the control group, which include those without a Lyme disease diagnosis or suspicion (Figure 6, “C” samples). Although *ospA* amplification was below 35 cycles in all Lyme disease samples (but none of the control samples), amplification with an average Cq below 35 cycles for both *Borrelia* genes (*ospA* and *flaB*) was obtained for only 12 of the 24 samples, corresponding to 9 of the 15 individuals diagnosed with Lyme disease. As culture serves to enrich the samples for *Borrelia*, we used the more conservative criterion of requiring amplification with both primer sets to mitigate the chance of false positives. Once more, the error bars were small. For the positive control (100 ng of *Borrelia* DNA), amplification was obtained with an average Cq of around 25 cycles for *ospA* and *flaB*. For the negative control of BSK-H cultures derived from the samples of individuals without a Lyme disease diagnosis or suspicion, no amplification was obtained below 35 cycles of amplification for either *ospA* or *flaB*.

Some study participants contributed only one sample, while others contributed multiple samples, at one time or at different times. For study participant L3, all three samples (L3g, L3p, and L3u) were positive for the detection of *Borrelia*. For the study participant # 102, both samples (L102g and L102u) were positive. For the study participant # 72, only one (L72g) of four samples (L72a, L72k1, L72k2, and L72g) was positive. For the study participants # 6, 35, 37, 70, 73, and 99, only one sample was tested per participant (L6p, L35g, L37g, L70g, L73g, and L99g) and they were positive. Samples of the study participants # 2, 5, 7, 12, 20, and 103 (L2g1, L2g2, L2g3, L5u, L5s, L7g, L12g, L20g, and L103g) were all considered negative. Using the same conservative criterion, we then compared the results based on the different sample types. Of the genital samples (n = 15), eight were considered positive for the presence of *Borrelia* DNA. Both periodontal samples and two of three urine samples were positive, whereas all three of the synovial fluid samples were negative.

### 3.3. Sanger Confirmation of Amplified Products

A cloned amplicon for each of the following biological sample types were sequenced: BSK-H cultures of the Lyme samples (L37g for *ospA* and L102g for *flaB*), ticks in the positive category (T299 for *ospA* and T383 for *flaB*), one tick in the negative category (T294 for *ospA* and *flaB*), and the *B. burgdorferi* control. Additionally, five *ospA* and one *flaB* larger amplicons were sequenced from the wildlife samples using the semi-nested PCR, eleven *ospA* amplicons and two *flaB* amplicons were obtained from ticks from the positive category were sequenced by two rounds of the conventional PCR, and seven *ospC* amplicons from BSK-H cultures were obtained with nested PCR. Table S1 provides the consensus sequence (forward and reverse combined) for each of these amplicons. A BLAST analysis of the NCBI GenBank database (see Supplementary Material, Table S2) confirmed that the primers are specifically targeting *Borrelia* with a 96.97% to 100% identity percentage to existing *Borrelia* GenBank sequences; the confirmation of the *Borrelia* strain, however, would require the sequencing of additional amplicons.

To further assess the amplicons of human origin (L3g, L3p, L35g, L70g, L72g, L73g, and L99g), a portion of the *Borrelia ospC* gene were sequenced and aligned to determine the *ospC* allele type. All of the sequences were identical (Supplemental Table S2). They were *ospC* A alleles, a globally-distributed allele that includes *B. burgdorferi* B31 which is of eastern North American origin [106,107]; however, these sequences were also 100% matches to natural isolates with *ospC* A alleles from western North America [108,109].

## 4. Discussion

### 4.1. Value of Optimized and Validated Tools for Direct Borrelia Detection

There is a need for optimized and validated tools to address both research and clinical problems associated with *Borrelia*, the causative agent of Lyme disease. Quantitative PCR is widely used in laboratories because it is a sensitive and specific fluorometric detection technique that allows for the real-time amplification, detection, and quantification of DNA or RNA without the need for post-PCR hands-on work, such as gel electrophoresis, to visualize results [72,73]. Furthermore, guidelines are available to standardize qPCR work (MIQE guidelines) [74], although it has been reported that most publications still do not provide enough details [75]. When carefully performed, qPCR is well-suited for the detection of infectious agents [76]. While the PCR approach has the advantage of directly detecting the pathogen, and so reducing ambiguity, it is not without its own limitations. Surveillance and clinical research applications have so far used a variety of primer sets, not always described sufficiently for replication, and optimization and validation are not always reported. Factors affecting *Borrelia* species detection by PCR include the sample types tested, the protocols used, and the course of pathogen infection [82]. In addition, the number of *Borrelia* bacteria, and so PCR targets, in clinical samples is lower than the number of targets for many other viral and bacterial infections [77,78,79,80,81], although the bacterial load of *Borrelia* in tick samples is much higher [83,84,85]. The low target abundance of *Borrelia* can be mitigated by the use of culture medium optimized for the growth of *Borrelia* [96,97]. PCR inhibitors in clinical samples, bacterial constituents, reagents, buffers, detergents, and antibiotics acting on nucleic acids or polymerases can cause false negatives. Reaction conditions can also interfere with PCR amplification if not optimized, leading to false negatives. Equally, cross-contamination can lead to false positives [91,92]. Nevertheless, there exist ways to mitigate these problems. False negatives from PCR inhibition can be detected using an internal control or can be avoided by using specific strategies to remove known PCR inhibitors [92], whereas false positives from cross-contamination can be prevented by good lab technique and the use of Uracil-N-glycosylase to degrade carryover PCR products in a preincubation step [93].

To optimize and fully describe the validation of such PCR tools was the motivation for this work. Our assay consists of a qPCR assay that targets two *Borrelia* genes (*ospA* and *flaB*), designed to qualitatively assess the presence or absence of *Borrelia* DNA in tick, wildlife, and culture-amplified human samples. While, in principle, any *Borrelia* genes could be used, *ospA* and *flaB*, a plasmid-borne gene and a chromosomal gene, respectively, have been used extensively as PCR targets, which is why we focused on these two genes. We found that different commercial qPCR master mixes affected the detection of *Borrelia* in our positive control (pure *Borrelia* DNA mixed in human DNA extracted from cell lines) to different extents; one was reliable for both primers, another generated results with a lower sensitivity, and the other did not work for either primer pair. Similarly, different commercial qPCR mixes have been shown to affect the sensitivity of detection of other DNA templates, including infectious agents [110,111]. The *ospA* primers seem to consistently show greater sensitivity than the *flaB* primers; nevertheless, both were effective in detecting sequence-validated *Borrelia* DNA in tick, wildlife, and culture-amplified human samples. For the samples with abundant *Borrelia* (tick and culture samples), we found that DNA isolation procedures did not greatly influence outcomes, but, in some cases, the optimization of DNA extraction is important. For the DNA extraction of most of our samples, we used methods that are known for producing a suboptimal DNA yield and sample purity. Nevertheless, they performed well, and they are a less expensive alternative to many commercial kits for DNA extraction. This is in accordance with the findings from the study by Okeyo and colleagues (2019) [112], in which the authors concluded that the variation in DNA yield from several extraction methods depended on the sample type (tissue or culture) as well as *Borrelia* DNA concentration. Thus, the use of the primers and protocols described here can be valuable in monitoring *Borrelia* in both tick vectors and in hosts.

### 4.2. Detection of Borrelia in Ticks

After optimization and validation, we applied our qPCR assay to detect *Borrelia* in ticks. The primers and protocol performed well, both confirming the results obtained by the nested PCR and clarifying the status of ticks with an “ambiguous” designation. The increased power of this approach, complemented by the reduced time and material handling required for qPCR relative to nested PCR, will be helpful in the detection of this pathogen in ticks. The quantitative PCR efficiency lowers after 30 cycles [113], so data should be interpreted carefully above that threshold. Using our positive control and our previously tested tick, wildlife, and human samples, we established criteria for samples to be considered as positive.

### 4.3. Detection of Borrelia in Wildlife

The direct detection of *Borrelia* in mammals, as opposed to ticks, remains a significant challenge due to the low copy number of *Borrelia* DNA within an overwhelming amount of mammalian DNA [101]. The bacterial load in ticks, wildlife tissues, and human tissues vary greatly depending on many, not all fully investigated, factors. The number of *Borrelia* spirochetes per tick have been estimated as varying between 10 to 10^6^, depending on whether the tick has fed, the time since feeding, the tick species, and the microbiome of the individual tick [85,114,115,116,117]. Wilhelmsson et al. [85] and Wang et al. [114] both reported an average of ~2000 *B. burgdorferi* spirochetes in unfed adult *I. scapularis* ticks, by direct counting and qPCR, respectively. This average is roughly comparable to the average *Borrelia* load per milligram of tissue of wildlife species [118]; however, the bacterial load varies appreciably depending on the wildlife species and tissue used [118], which, combined with the larger mammalian genome, makes the detection of *Borrelia* in mammalian tissues more technically challenging. To determine if our qPCR protocol could directly detect *Borrelia* DNA in mammalian wild reservoir species, we tested DNA samples from different species previously shown to be infected with *Borrelia*, or not, by nested PCR [101]. While the sensitivity of the qPCR protocol in the absence of a pre-amplification step was low (10%), upon pre-amplification, the *ospA* primers worked well in amplifying the product from some of the positive samples. The other samples may have suffered DNA degradation during storage, or been below the limit of detection, or were false positives when assessed by the nested PCR. The bacteria might also have been located in tissues other than the ones tested. The *ospA* primer set also amplified the product, which were sequence-confirmed as *B. burgdorferi*, from four of the samples previously categorized as negative. The identification, with sequence confirmation, of four samples (C3K16, C70K16, C94K16, and C6K17) previously considered uninfected by the nested PCR [101] may indicate that the protocol used here is more sensitive than the nested protocol. Further work would be needed to determine the sensitivity and specificity relative to other PCR protocols. Consistent with the lower sensitivity of the *flaB* primer set, these primers generated non-specific amplicons from the wildlife samples. This indicates that, while this primer set is effective with samples with abundant *Borrelia* target DNA such as tick or culture-amplified samples, these primers are not specific enough for use with wild mammal samples.

### 4.4. Detection of Borrelia in Culture-Amplified Human Lyme Cultures

The direct detection of *Borrelia* in humans, who are accidental rather than reservoir hosts for *Borrelia*, remains a significant challenge due to the low number of *Borrelia* cells in human tissues [77,78,79,80,81,119]. Schmidt estimates that the higher end of the *Borrelia* burden in humans is approximately 4000 spirochetes/gram of plasma, and notes that this value will be lower in chronic/late infections [80]. Assuming the average DNA recovery from blood (Qiagen) [120], this corresponds to a maximum of 0.1 *Borrelia* spirochetes per ng of human DNA, approximately three to four orders of magnitude lower than wildlife [118]. Culture addresses this problem by amplifying *Borrelia* from biological tissue or fluid samples [99]. Culturing samples from people diagnosed with Lyme disease provides a better chance at detecting *Borrelia* than testing the sample without cultivation; however, using a BSK-H culture does preclude the quantification of the bacterial load in the original sample and the culturing process delays the obtaining of results as *Borrelia* are slow-growing bacteria [121,122,123,124]. The BSK-H cultures sent by our clinical partners were derived from different sample types. Based on the nature and course of an infection with *Borrelia* species, some of these samples might not be ideal and the tissue distribution in late-stage Lyme disease has been understudied [80]. For example, *B. burgdorferi* does not stay in the bloodstream for long and undergoes only sporadic bursts of spirochetemia [125]. Furthermore, *Borrelia* is known to infect many tissues and its presence in genital fluids has been reported [102] but remains controversial. The isolation of *Borrelia* from genital fluids could arise from cross-contamination with blood or urine or from infection of the gonads and accessory tissues. Bosler and Schulze (1986) [126] report that *Borrelia* can be cultured from the urine of the wild reservoir host, *Peromyscus leucopus*, although Wright and Nielsen (1990) [127] were not able to find microscopic evidence of spirochaetes in mouse urine. Similarly, live spirochaetes have been reported to have been cultured from urine from cows (Burgess 1988) [128]. Culture from canine urine has been found by some researchers (Grauer et al. 1988) [129] but not others (Appel et al. 1993) [130]. While many of the samples tested here were unconventional, they do have the advantage of avoiding invasive sample collection. Using different sample types and biological replicates (same sample types from the same patient collected at different times), as included in this study, could increase the chance of collecting samples at the appropriate time and place during the course of infection and, thus, avoiding false negative results.

*Borrelia* DNA was not found in all of the cultures from individuals with a diagnosis or suspicion of Lyme disease. In this small sample, there seemed to be a poor correlation between the serology (when available) and PCR analysis of cultures from the participants; of the three with a positive serology (L2, L5, and L6), only one was positive by PCR, and, of the five with an indeterminate or negative serology (L3, L7, L12, L20, and L35), two were positive by PCR. The complex nature of the course of infection and flaws in standard serological testing can lead to delayed, failed, or erroneous diagnoses, in many cases, over the course of years [53,54,55,56,57,58,59,60,61]. As is true of all tests dependent on antibody detection, the results of Lyme disease testing are undesirably inaccurate in some cases [64,71]. Possible reasons for the non-detection of *Borrelia* in samples from seropositive individuals might be the absence of *Borrelia* in that fluid or tissue at the time the sample was taken or a resolved infection. Possible explanations for positive PCR findings in samples from seronegative individuals could be the insensitivity of the serological diagnosis, misdiagnosis of the individual, or contamination. Contamination is always a concern that needs to be addressed when using protocols that amplify biological agents, in this case, both culture and PCR. Opportunities for the contamination of the cultures were minimal in this study. The culture medium was commercially supplied, and, while aliquoted prior to sample collection, there was no pattern of consecutive samples being positive as might happen if a series of culture tubes were contaminated in preparation. The cultures were generally inoculated by the participants in the participants’ homes, locations not expected to have access to pure cultures of *Borrelia*. Once inoculated, the culture tubes remained sealed until DNA isolation was performed. DNA isolation and PCR were performed in different buildings, and both the biological and technical negative controls were negative, reducing the likelihood of DNA contamination. The *ospC* genotyping of DNA from these culture-amplified samples showed that they all had identical type A *ospC* alleles (Supplemental Table S2), which may not be surprising as the most common and globally distributed allele found in both eastern and western North America. The absence of full serological and medical assessments of the human participants in this portion of the study and the non-linear *Borrelia* amplification by culture precludes the determination of the sensitivity or specificity of this protocol. Nevertheless, there is value in methods that are applicable to “real-world” situations and this protocol did detect *Borrelia* DNA in half of the samples, which corresponds to 60% of individuals with a diagnosis or suspicion of Lyme disease.

Tools such as we describe here will aid in tick surveillance and wildlife surveillance, and to understand the mechanism of pathogenesis in humans. Lyme disease is considered a climate disease [6,7,8,9,10,131,132], which indicates that the prevalence, and cost in both human suffering and at the societal level, has been increasing and will continue to do so. Well-validated tools for direct detection provide one tool to support the surveillance and research in ticks, reservoir hosts, and accidental hosts such as companion animals, agricultural animals, and humans.

## Figures and Tables

**Table 1:** *Ixodes scapularis* ticks collected by passive surveillance in 2020 and *Borrelia* infection status.

Tick ID	Collection Date	Collection Location	Engorgement Status	Sex and Life Cycle	Host	Tick Attachment Status	*Borrelia burgdorferi* Testing ^1^
T248	1 October	NB, CA	Engorged	Adult female	*Canis familiaris*	Attached	+
T249	1 October	NB, CA	Engorged	Adult female	*Canis familiaris*	Attached	+
T260	16 October	NB, CA	Highly engorged	Adult female	*Canis familiaris*	Attached	+
T264	21 October	NB, CA	Highly engorged	Adult female	*Canis familiaris*	Attached	+
T266	19 October	NB, CA	Engorged	Adult female	*Canis familiaris*	Attached	+
T282	1 November	NB, CA	Highly engorged	Adult female	*Canis familiaris*	Attached	+
T285	10 October	NB, CA	Highly engorged	Adult female	*Canis familiaris*	Attached	+
T297	October ^2^	NB, CA	Engorged	Adult female	*Felis catus*	Attached	+
T299	2 November	NB, CA	Highly engorged	Adult female	*Canis familiaris*	Attached	+
T306	29 October	NB, CA	Engorged	Adult female	*Canis familiaris*	Unattached	+
T326	10 November	NS, CA	Engorged	Adult female	*Homo sapiens*	Attached	+
T375	14 November	NB, CA	Highly engorged	Adult female	*Canis familiaris*	Attached	+
T383	21 November	NS, CA	Engorged	Adult female	*Homo sapiens*	Attached	+
T388	16 November	NS, CA	Highly engorged	Adult female	*Homo sapiens*	Attached	+
T389	16 November	NS, CA	Engorged	Adult female	*Homo sapiens*	Attached	+
T408	21 November	NB, CA	Engorged	Adult female	*Equus caballus*	Attached	+
T413	18 November	NB, CA	Engorged	Adult female	*Canis familiaris*	Attached	+
T424	18 November	NB, CA	Engorged	Adult female	*Canis familiaris*	Attached	+
T440	2 December	NB, CA	Engorged	Adult female	*Canis familiaris*	Unknown ^2^	+
T441	2 December	NB, CA	Engorged	Adult female	*Canis familiaris*	Unknown ^2^	+
T221	30 July	PE, CA	Engorged	Adult female	*Felis catus*	Attached	-
T237	23 May	NB, CA	Engorged	Adult female	*Homo sapiens*	Attached	-
T243	30 July	NB, CA	Engorged	Adult female	*Canis familiaris*	Attached	-
T252	30 September	NB, CA	Engorged	Adult female	*Canis familiaris*	Attached	-
T298	October ^2^	NB, CA	Highly engorged	Adult female	*Felis catus*	Attached	-
T302	27 October	PE, CA	Engorged	Adult female	*Canis familiaris*	Attached	-
T303	27 October	PE, CA	Engorged	Adult female	*Canis familiaris*	Attached	-
T390	14 November	NS, CA	Engorged	Adult female	*Homo sapiens*	Attached	-
T407	24 November	NB, CA	Engorged	Adult female	*Canis familiaris*	Attached	-
T414	21 November	NB, CA	Engorged	Adult female	*Canis familiaris*	Attached	-
T238	14 September	NB, CA	Highly engorged	Adult female	*Bos taurus*	Attached	Ambiguous
T248	1 October	NB, CA	Engorged	Adult female	*Canis familiaris*	Attached	Ambiguous
T249	1 October	NB, CA	Engorged	Adult female	*Canis familiaris*	Attached	Ambiguous
T260	16 October	NB, CA	Highly engorged	Adult female	*Canis familiaris*	Attached	Ambiguous
T285	10 October	NB, CA	Highly engorged	Adult female	*Canis familiaris*	Attached	Ambiguous
T294	24 October	NB, CA	Highly engorged	Adult female	*Canis familiaris*	Attached	Ambiguous
T359	13 November	PE, CA	Engorged	Adult female	*Canis familiaris*	Attached	Ambiguous
T415	24 November	PE, CA	Engorged	Adult female	*Canis familiaris*	Attached	Ambiguous
T423	28 November	NB, CA	Engorged	Adult female	*Canis familiaris*	Attached	Ambiguous
T424	18 November	NB, CA	Engorged	Adult female	*Canis familiaris*	Attached	Ambiguous

^1^ *B. burgdorferi* testing is based on the nested PCR results of the article by Lewis et al. (2021) [100]. ^2^ Information from the donor was incomplete or unknown. NB = New Brunswick; NS = Nova Scotia; PE = Prince Edward Island; CA = Canada.

**Table 2:** Wildlife samples collected between 2016 and 2017 and *Borrelia* infection status.

Sample ID ^1^	Year of Collection	Source for the Collection	Species	Type of Tissue	*Borrelia burgdorferi* Testing ^1^
C3K16	2016	Cat kill	*Microtus pennsylvanicus*	Kidney	-
C9K16	2016	Cat kill	*Microtus pennsylvanicus*	Kidney	-
C13K16	2016	Cat kill	*Microtus pennsylvanicus*	Kidney	-
C30K16	2016	Cat kill	*Peromyscus maniculatus*	Kidney	-
C50K16	2016	Cat kill	*Microtus pennsylvanicus*	Kidney	-
C54K16	2016	Cat kill	*Microtus pennsylvanicus*	Kidney	-
C70K16	2016	Cat kill	*Microtus pennsylvanicus*	Kidney	-
C94K16	2016	Cat kill	*Microtus pennsylvanicus*	Kidney	-
C99K16	2016	Cat kill	*Microtus pennsylvanicus*	Kidney	-
C113L16	2016	Cat kill	*Acanthis flammea*	Liver	-
C126K16	2016	Cat kill	*Microtus pennsylvanicus*	Kidney	-
C130K16	2016	Cat kill	*Microtus pennsylvanicus*	Kidney	-
C6K17	2017	Cat kill	*Zapus hudsonius*	Kidney	-
C6L17	2017	Cat kill	*Zapus hudsonius*	Liver	-
R1K16	2016	Roadkill	*Erethizon dorsatum*	Liver	+
R1L16	2016	Roadkill	*Zapus hudsonius*	Liver	+
R6L16	2016	Roadkill	*Corvus brachyrhynchos*	Liver	+
C5L16	2016	Cat kill	*Microtus pennsylvanicus*	Liver	+
C6K16	2016	Cat kill	*Zapus hudsonius*	Kidney	+
C8K16	2016	Cat kill	*Zapus hudsonius*	Kidney	+
C50k17	2017	Cat kill	*Blarina brevicauda*	Kidney	+

^1^ Information on samples and *B. burgdorferi* testing by nested PCR are as described in Zinck and Lloyd (2022) [101].

**Table 3:** Human samples information and results of previous *Borrelia* serological testing.

Donor Code ^1^	Clinical Diagnosis ^2^	Age ^3^	Biological Sex	Lyme Serology ^2^	Type of Sample	Sample Code ^1^
L2	Lyme	62	F	IgM pos	Genital sample	L2g1
				IgG neg	Genital sample	L2g2
					Genital sample	L2g3
L3	Lyme	65	F	IFA Ind WB neg	Genital sample	L3g
					Periodontal sample	L3p
					Urine sample	L3u
L5	No diagnosis	75	F	WB Pos	Skin sample	L5s
					Urine sample	L5u
L6	No diagnosis	44	M	WB Pos	Periodontal sample	L6p
L7	Lyme	50	M	WB Ind	Genital sample	L7g
L12	Lyme	54	F	WB Ind	Genital sample	L12g
L20	Suspicion	63	F	WB neg	Genital sample	L20g
L35	Suspicion	64	F	WB neg	Genital sample	L35g
L37	No diagnosis	Undisclosed ^4^	F	Untested	Genital sample	L37g
L70	Suspicion	80	F	Untested	Genital sample	L70g
	Suspicion	58	F	Untested	Ankle synovial fluid	L72a
					Genital sample	L72g
					Knee synovial fluid	L72k1
					Knee synovial fluid	L72k2
L73	Suspicion	63	M	Untested	Seminal liquid sample	L73g
L99	No diagnosis	41	F	Untested	Genital sample	L99g
L102	No diagnosis	53	F	Untested	Genital sample	L102g
					Urine sample	L102u
L103	No diagnosis	38	M	Untested	Genital sample	L103g
C9	Healthy	30	F	Untested	Blood sample	C9b
C23	Healthy	59	F	Untested	Blood sample	C23b
C64	Healthy	59	F	Untested	Blood sample	C64b
C3	Healthy	29	F	Untested	Genital sample	C3g
C25	Healthy	59	F	Untested	Genital sample	C25g
C84	Healthy	60	F	Untested	Genital sample	C84g

^1^ Samples starting with the letter “L” are Lyme samples and samples starting with the letter “C” are control samples (samples of individual without a Lyme diagnosis or a suspicion of Lyme disease). Samples ending with the letter “g” are genital samples, samples ending with the letter “p” are periodontal samples, samples ending with the letter “u” are urine samples, sample ending with the letter “s” is a skin sample, sample ending with the letter “a” is a sample of ankle synovial fluid, samples ending with the letter “k” are samples of knee synovial fluid, and samples ending with the letter “b” are blood samples. Numbers at the end of some of the samples’ names (1, 2, or 3) indicate the biological replicates. ^2^ Suspicion indicates a clinical suspicion of Lyme disease but no formal diagnosis. Serology was obtained from different clinical laboratories. Ind = indeterminate serology, WB = Western blot, IFA = immuno-fluorescent assay. ^3^ Age refers to age at the time of sample donation. ^4^ Information not provided by participant.

**Table 5:** Optimization of the DNA extraction method using *Borrelia burgdorferi* B31 pure culture.

Kit or Solution Used	Average DNA Concentration (ng/µL) ^1^	Extraction Volume (µL)	Average Quantity (ng) ^1^
AquaGenomic solution (MultiTarget Pharmaceuticals LLC)	17.40 +/− 23.06	50	870 +/− 758.75
DNeasy Blood & Tissue kits (Qiagen)	2.12 +/− 1.06	200	423.3 +/− 163.3
PureLink Genomic DNA kits (Thermo Fisher Scientific)	12.17 +/− 7.86	50	608.3 +/− 294.4
Monarch Genomic DNA purification kit (New England Biolabs)	4.7 +/− 2.45	100	470 +/− 180
Extracta DNA prep (Quantabio)	114.4 +/− 21.02	200	22,876.7 +/− 3271.1

^1^ Average DNA concentration and quantity, and standard deviation (indicated by “+/−”), was measured using the individual DNA extraction of 6–8 samples of *B. burgdorferi* B31 pure cultures, using the nanodrop ND-1000.

**Table 6:** Result for the PCR testing on wildlife samples.

ID	Tissue	Species	Nested PCR ^1^	Average Cq of qPCR without Pre-Amplification		Average Cq of qPCR with Pre-Amplification		Two Rounds of Conventional PCR ^2^		Semi-Nested PCR ^2^	
				*ospA*	*flaB*	*ospA*	*flaB*	*ospA*	*flaB*	*ospA*	*flaB*
C3K16	*Microtus pennsylvanicus*	Kidney	-	22.6	23.2	19.3	-	-	-	+	-
C9K16	*Microtus pennsylvanicus*	Kidney	-	-	-	-	-	Non- specific	-	No BLAST result ^3^	-
C13K16	*Microtus pennsylvanicus*	Kidney	-	-	33.7	-	-	Non- specific	-	-	-
C30K16	*Peromyscus maniculatus*	Kidney	-	-	34.3	-	-	Non- specific	-	-	-
C50K16	*Microtus pennsylvanicus*	Kidney	-	-	-	-	-	-	Non- specific	-	-
C54K16	*Microtus pennsylvanicus*	Kidney	-	-	-	-	-	Non- specific	Non- specific	-	-
C70K16	*Microtus pennsylvanicus*	Kidney	-	-	-	31.3	-	-	-	+	-
C94K16	*Microtus pennsylvanicus*	Kidney	-	-	-	28.3	-	Non- specific	-	+	-
C99K16	*Microtus pennsylvanicus*	Kidney	-	-	-	-	-	Non- specific	-	-	-
C113L16	*Acanthis flammea*	Liver	-	-	-	-	-	-	-	-	-
C126K16	*Microtus pennsylvanicus*	Kidney	-	-	-	-	39.1	Non- specific	-	-	-
C130K16	*Microtus pennsylvanicus*	Kidney	-	-	-	-	-	Non- specific	-	-	-
C6K17	*Zapus hudsonius*	Kidney	-	-	-	23.9	-	-	-	+	+
C6L17	*Zapus hudsonius*	Liver	-	-	-	-	-	-	-	-	-
R1K16	*Erethizon dorsatum*	Liver	+	-	-	30.9	-	No BLAST result ^3^	-	-	-
R1L16	*Zapus hudsonius*	Liver	+	-	-	27.3	-	-	-	+	-
R6L16	*Corvus brachyrhynchos*	Liver	+	-	-	39.2	-	-	-	No BLAST result ^3^	-
C5L16	*Microtus pennsylvanicus*	Liver	+	-	-	-	-	No BLAST result ^3^	-	-	-
C6K16	*Zapus hudsonius*	Kidney	+	-	-	-	-	No BLAST result ^3^	-	-	-
C8K16	*Zapus hudsonius*	Kidney	+	-	-	-	-	-	-	-	-
C50k17	*Blarina brevicauda*	Kidney	+	-	-	-	30.8	-	-	No BLAST result ^3^	No BLAST result ^3^

^1^ Results are taken from the article by Zinck and Lloyd (2022) [101], in Table S2. ^2^ The minus sign (-) represents the absence of an amplicon and the plus sign (+) represents the presence of a sequence-confirmed *Borrelia* amplicon. ^3^ BLAST result was not obtained due to a lack of similarities found between our amplified sequences and the BLAST databank sequences or amplicons with poor sequence quality.

## Data Availability

The original contributions presented in this study are included in the article/Supplementary Materials. Further inquiries can be directed to the corresponding authors.

## Supplementary Materials

The following supporting information can be downloaded at: https://www.mdpi.com/article/10.3390/pathogens13121034/s1, Table S1: Sequences of amplicons obtained from Sanger sequencing; Table S2: BLAST analysis of amplicons sequenced; Figure S1: Gel electrophoresis of semi-nested amplicons from human tissues samples.
