# Supplementary material for: Development, Optimization, and Validation of a Quantitative PCR Assay for Borrelia burgdorferi Detection in Tick, Wildlife, and Human Samples
Source: Pathogens. 2024 Nov 23;13(12):1034. doi: 10.3390/pathogens13121034 (PMC11679815; doi:10.3390/pathogens13121034)
Supplement: Supplementary file 1 [file pathogens-13-01034-s001.zip › pathogens-3281329-supplementary.pdf]

**Supplementary materials**

**Table S1**. Sequences of amplicons obtained from Sanger sequencing.

| Type of PCR | Targeted gene | Samples | Consensus sequence (5′ → 3′) |
|---|---|---|---|
| Real-time PCR | *ospA* | T294 | TGGCGGCCGCGGGAATTCGATTGAACCAGACTTGAATACACAGGAATTAAAAGCGATGGATCCGGAAAAGCTAAAGAGGTTTTAAAAGGCTATGTTCTTGAAGGAACTCTAACTGCTGAAAATCACTAGTGAATTCGCGGCCGCCTGCAGGTCGACCATATGGGAGAGCTC |
| | | T299 | TGGCGGCCGCGGGAATTCGATTGAACCAGACTTGAATACACAGGAATTAAAAGCGATGGATCCGGAAAAGCTAAAGAGGTTTTAAAAGGCTATGTTCTTGAAGGAACTCTAACTGCTGAAAATCACTAGTGAATTCGCGGCCGCCTGCAGGTCGACCATATGGGAGAGCTCCCAACGCGTT |
| | | L37g | CGCGAATTCACTAGTGATTGAACCAGACTTGAATACACAGGAATTAAAAGCGATGGATCTGGAAAAGCTAAAGAGGTTTTAAAAGGCTATGTTCTTGAAGGAACTCTAACTGCTGAAAATCGAATTCCCGC |
| | | *B. burgdorferi* | GCCGCGAATTCACTAGTGATTTTCAGCAGTTAGAGTTCCTTCAAGAATATAGCCTTTTAAAACCTCTTTAGCTTTTCCAGATCCATCGCTTTTAATTCCTGTGTATTCAAGTCTGGTTCAATCGAATTCCC |
| | *flaB* | T294 | ATGGCGGCCGCGGGAATTCGATTCAATCAGGTAACGGCACATATTCAGATGCAGACAGAGGTTCTATACAAATTGAAATAGAGCAACTTACAGACGAAATTAATAGAATCACTAGTGAATTCGCGGCCGCCTGCAGGTCGACCATATGGGAGAGCTCCCAA |
| | | T383 | GGCGGCCGCGGGAATTCGATTCTATTAATTTCGTCTGTAAGTTGCTCTATTTCAATTTGTATAGAACCTCTGTCTGCATCTGAATATGTGCCGTTACCTGATTGAATCACTAGTGAATTCGCGGCCGCCTGCAGGTCGACCATATGGGAGAGCTCCCAACGCGTTGGATGC |
| | | L102g | CCGCGAATTCACTAGTGATTCAATCAGGTAACGGCACATATTCAGATGCAGACAGAGGTTCTATACAAATTGAAATAGAGCAACTTACAGACGAAATTAATAGAATCGAATTCCCGCGGCCGCCATGGCGG |
| | | *B. burgdorferi* | CCGCGAATTCACTAGTGATTCAATCAGGTAACGGCACATATTCAGATGCAGACAGAGGTTCTATACAAATTGAAATAGAGCAACTTACAGACGAAATTAATAGAATCGAATTCCCGCGGCCGCCATGGCGG |
| Semi-nested PCR | *ospA* | C3K16 | CTGGAAAAGCTAAAGAGGTTTTAAAAGGCTATGTTCTTGAAGGAACTTTAACTGCTGAAAAAACAACCTTGGTGGTTAAAGAAGGAACTGTTACTTTAAGCAAAAA |
| | | C6K17 | GGATCTGGAAAAGCTAAAGAGGTTTTAAAAGGCTATGTTCTTGAAGGAACTCTAACTGCTGAAAAAACAACATTGGTGGTAAAGAAGGAACTGTA |
| | | C70K16 | CTGGAAAAGCTAAAGAGGTTTTAAAAGGCTATGATTCTTGAAGGAACTCTAACTGCTGAAAAAACAACATTGGTGGTAAAGAAGGAA |
| | | C9K16 | CTGGAAAAGCTAAAGAGGTTTTAAAAGGCTATGTTCTTGAAGGAACTCTAACTGCTGAAAAAACAACATTGGGGTTAAGAAGGAACTG |

| | | | |
|---|---|---|---|
| | | R1L16 | GAAGGAACTCTACCTGCTGAAAAAACACCTTTGGTGGTTAAAGAAGGAACTGTTTACTTTA AGCAAAAATATTTCAAAATCTGGGGAAGTTTCAGTTGAA |
| | *flaB* | C6K17 | GCTTAAAGTAACAGTTCCTTCTTTACCACCAATGTTGTTTTTTCAGCAGTTAGAGTTCCTT CAAGAACATAGCC TTTTAAAACCTCTTTAGCTTTTCC |
| 2 rounds of amplification with outer primers | *ospA* | T299 | ATTAAAAGCGATGGATCCGGAAAAGCTAAAGAGGTTTTAAAAGGCTATGTTCTTGAAGGAA CTCTAACTGCTGAAAAAACAACATTGGTGGTAAAGAAGGAACTGTACTT |
| | *flaB* | T299 | TATTCAGATGCAGACAGAGGTTCTATACAAATTGAAATAGAGCAACTTACAGACGAAATTA ATAGAATTGCTGATCAAGCTCAATATAACCAAATGC |
| | | T375 | GGTTCTATACAAATTGAAATAGAGCAACTTACAGACGAAATTAATAGAATTGCTGATCAAG CCCAATATAACCAAATGCACATGTTATCAAACAAATCTGCTTCTCA |
| Nested PCR | *ospC* | L3g | TCTATAGATGAAATTGCTGCTAAAGCTATTGGTAAAAAAATACACCAAAATAATGGTTTGG ATACCGAAAATAATCACAATGGATCATTGTTAGCGGGAGCTTATGCAATATCAACCCTAAT AAAACAAAAATTAGATGGATTGAAAAATGAAGGATTAAAGGAAAAAATTGATGCGGCTAA GAAATGTTCTGAAACATTTACTAATAAATTAAAAGAAAAACACA |
| | | L3p | TCTATAGATGAAATTGCTGCTAAAGCTATTGGTAAAAAAATACACCAAAATAATGGTTTGG ATACCGAAAATAATCACAATGGATCATTGTTAGCGGGAGCTTATGCAATATCAACCCTAAT AAAACAAAAATTAGATGGATTGAAAAATGAAGGATTAAAGGAAAAAATTGATGCGGCTAA GAAATGTTCTGAAACATTTACTAATAAATTAAAAGAAAAAC |
| | | L35g | GCTATTGGTAAAAAAATACACCAAAATAATGGTTTGGATACCGAAAATAATCACAATGGAT CATTGTTAGCGGGAGCTTATGCAATATCAACCCTAATAAAACAAAAATTAGATGGATTGAA AAATGAAGGATTAAAGGAAAAAATTGATGCGGCTAAGAAATGTTCTGAAACATTTACTAAT AAATTAAAAGAAAAACACACAGATC |
| | | L70g | AAGCTATTGGTAAAAAAATACACCAAAATAATGGTTTGGATACCGAAAATAATCACAATG GATCATTGTTAGCGGGAGCTTATGCAATATCAACCCTAATAAAACAAAAATTAGATGGATT GAAAAATGAAGGATTAAAGGAAAAAATTGATGCGGCTAAGAAATGTTCTGAAACATTTAC TAATAAATTAAAAGAAAAACACACA |
| | | L72g | CTATAGATGAAATTGCTGCTAAAGCTATTGGTAAAAAAATACACCAAAATAATGGTTTGGA TACCGAAAATAATCACAATGGATCATTGTTAGCGGGAGCTTATGCAATATCAACCCTAATA AAACAAAAATTAGATGGATTGAAAAATGAAGGATTAAAGGAAAAAATTGATGCGGCTAAG AAATGTTCTGAAACATTTACTAATAAATTAAAAGAAAAACACACAGATC |
| | | L73g | TCTATAGATGAAATTGCTGCTAAAGCTATTGGTAAAAAAATACACCAAAATAATGGTTTGG ATACCGAAAATAATCACAATGGATCATTGTTAGCGGGAGCTTATGCAATATCAACCCTAAT AAAACAAAAATTAGATGGATTGAAAAATGAAGGATTAAAGGAAAAAATTGATGCGGCTAA GAAATGTTCTGAAACATTTACTAATAAATTAAAAGAAAAACACACAGATCT |
| | | L99g | CTATAGATGAAATTGCTGCTAAAGCTATTGGTAAAAAAATACACCAAAATAATGGTTTGGA TACCGAAAATAATCACAATGGATCATTGTTAGCGGGAGCTTATGCAATATCAACCCTAATA AAACAAAAATTAGATGGATTGAAAAATGAAGGATTAAAGGAAAAAATTGATGCGGCTAAG AAATGTTCTGAAACATTTACTAATAAATTAAAAGAAAAACACACAGATC |

**Table S2**. BLAST analysis of amplicons sequenced.

| Type of PCR | Targeted gene | Sample name | BLAST result position | Description | Maximal score | Total score | Query percentage (%) | E-value | Identity percentage (%) |
|---|---|---|---|---|---|---|---|---|---|
| Real-time PCR | *ospA* | T294 | 1 | *Borrelia burgdorferi* (GenBank # GQ247743.1) | 185 | 185 | 58% | 1e-42 | 100% |
| | | | 2 | *Borrelia burgdorferi* (GenBank # AF309007.1) | 185 | 185 | 58% | 1e-42 | 100% |
| | | | 3 | *Borrelia burgdorferi* (GenBank # L23144.1) | 185 | 185 | 58% | 1e-42 | 100% |
| | | T299 | 1 | *Borrelia burgdorferi* (GenBank # GQ247743.1) | 185 | 185 | 55% | 1e-42 | 100% |
| | | | 2 | *Borrelia burgdorferi* (GenBank # AF309007.1) | 185 | 185 | 55% | 1e-42 | 100% |
| | | | 3 | *Borrelia burgdorferi* (GenBank # L23144.1) | 185 | 185 | 55% | 1e-42 | 100% |
| | | L37g | 1 | *Borrelia* species (GenBank # KY328701.1) | 185 | 185 | 76% | 9e-43 | 100% |
| | | | 2 | *Borrelia burgdorferi* (GenBank # CP019925.1) | 185 | 185 | 76% | 9e-43 | 100% |
| | | | 3 | *Borrelia burgdorferi* (GenBank # CP019854.1) | 185 | 185 | 76% | 9e-43 | 100% |
| | | *B. burgdorferi* B31 | 1 | *Borrelia* species (GenBank # KY328701.1) | 180 | 180 | 76% | 4e-41 | 99% |
| | | | 2 | *Borrelia burgdorferi* (GenBank # CP019925.1) | 180 | 180 | 76% | 4e-41 | 99% |
| | | | 3 | *Borrelia burgdorferi* (GenBank # CP019854.1) | 180 | 180 | 76% | 4e-41 | 99% |
| | *flaB* | T294 | 1 | *Borrelia burgdorferi* (GenBank # KR782216.1) | 165 | 165 | 52% | 2e-36 | 96.97% |
| | | | 2 | *Borrelia carolinensis* (GenBank # MK604308.1) | 165 | 165 | 51% | 6e-36 | 96.97% |
| | | | 3 | *Borrelia* species (GenBank # MH807157.1) | 165 | 165 | 51% | 6e-36 | 96.97% |

| | | | | | | | | |
|---|---|---|---|---|---|---|---|---|
| | T383 | 1 | *Borrelia burgdorferi* (GenBank # KR782216.1) | 165 | 165 | 67% | 1e-36 | 100% |
| | | 2 | *Borrelia carolinensis* (GenBank # MK604308.1) | 163 | 163 | 67% | 4e-36 | 100% |
| | | 3 | *Borrelia* species (GenBank # MH807157.1) | 163 | 163 | 67% | 4e-36 | 100% |
| | L102g | 1 | *Borrelia burgdorferi* (GenBank # KR782216.1) | 165 | 165 | 67% | 1e-36 | 100% |
| | | 2 | *Borrelia carolinensis* (GenBank # MK604308.1) | 163 | 163 | 67% | 4e-36 | 100% |
| | | 3 | *Borrelia* species (GenBank # MH807157.1) | 163 | 163 | 67% | 4e-36 | 100% |
| | *B. burgdorferi* B31 | 1 | *Borrelia burgdorferi* (GenBank # KR782216.1) | 165 | 165 | 67% | 1e-36 | 100% |
| | | 2 | *Borrelia carolinensis* (GenBank # MK604308.1) | 163 | 163 | 67% | 4e-36 | 100% |
| | | 3 | *Borrelia* species (GenBank # MH807157.1) | 163 | 163 | 67% | 4e-36 | 100% |
| Semi-nested *ospA* PCR | C3K16 | 1 | *Borrelia burgdorferi* (GenBank # CP161918.1) | 191 | 191 | 100% | 1e-44 | 99.06% |
| | | 2 | *Borrelia burgdorferi* (GenBank # GU826945.1) | 191 | 191 | 100% | 1e-44 | 99.06% |
| | | 3 | *Borrelia burgdorferi* (GenBank # KM069287.1) | 191 | 191 | 100% | 1e-44 | 99.06% |
| | C6K17 | 1 | *Borrelia* species (GenBank # MN461277.1) | 169 | 169 | 98% | 6e-38 | 98.95% |
| | | 2 | *Borrelia burgdorferi* (GenBank # KM069285.1) | 169 | 169 | 98% | 6e-38 | 98.95% |
| | | 3 | *Borrelia burgdorferi* (GenBank # CP019925.1) | 169 | 169 | 98% | 6e-38 | 98.95% |
| | C70K16 | 1 | *Borrelia* species (GenBank # MN461277.1) | 150 | 150 | 100% | 2e-32 | 97.73% |
| | | 2 | *Borrelia burgdorferi* (GenBank # KM069285.1) | 150 | 150 | 100% | 2e-32 | 97.73% |
| | | 3 | *Borrelia burgdorferi* (GenBank # CP019925.1) | 150 | 150 | 100% | 2e-32 | 97.73% |
| | C9K16 | 1 | *Borrelia* species (GenBank # MN461277.1) | 154 | 154 | 100% | 1e-33 | 97.78% |

| | | | | | | | | | |
|---|---|---|---|---|---|---|---|---|---|
| | | | 2 | *Borrelia burgdorferi* (GenBank # KM069285.1) | 154 | 154 | 100% | 1e-33 | 97.78% |
| | | | 3 | *Borrelia burgdorferi* (GenBank # CP019925.1) | 154 | 154 | 100% | 1e-33 | 97.78% |
| | | R1L16 | 1 | *Borrelia burgdorferi* (GenBank # GQ443125.1) | 161 | 161 | 100% | 1e-35 | 96% |
| | | | 2 | *Borrelia burgdorferi* (GenBank # AF369937.1) | 161 | 161 | 100% | 1e-35 | 96% |
| | | | 3 | *Borrelia burgdorferi* (GenBank # KM069285.1) | 161 | 161 | 100% | 1e-35 | 96% |
| | *flaB* | C6K17 | 1 | *Borrelia burgdorferi* (GenBank # GQ443125.1) | 176 | 176 | 100% | 4e-40 | 98.99% |
| | | | 2 | *Borrelia* species (GenBank # MN461277.1) | 176 | 176 | 100% | 4e-40 | 98.99% |
| | | | 3 | *Borrelia burgdorferi* (GenBank # KM069285.1) | 176 | 176 | 100% | 4e-40 | 98.99% |
| 2 rounds of amplification with outer primers | *ospA* | T299 | 1 | *Borrelia burgdorferi* (GenBank # GQ443125.1) | 195 | 195 | 100% | 1e-45 | 98.21% |
| | | | 2 | *Borrelia burgdorferi* (GenBank # CP001421.1) | 195 | 195 | 100% | 1e-45 | 98.21% |
| | | | 3 | *Borrelia burgdorferi* (GenBank # GQ247743.1) | 195 | 195 | 100% | 1e-45 | 98.21% |
| | *flaB* | T299 | 1 | *Borrelia lusitaniae* (GenBank # PP938597.1) | 180 | 180 | 100% | 3e-41 | 100% |
| | | | 2 | *Borrelia burgdorferi* (GenBank # AF264885.1) | 180 | 180 | 100% | 3e-41 | 100% |
| | | | 3 | *Borrelia burgdorferi* (GenBank # KC246023.1) | 180 | 180 | 100% | 3e-41 | 100% |
| | | T375 | 1 | *Borrelia burgdorferi* (GenBank # CP161091.1) | 198 | 198 | 100% | 9e-47 | 100% |
| | | | 2 | *Borrelia burgdorferi* (GenBank # MT039712.1) | 198 | 198 | 100% | 9e-47 | 100% |
| | | | 3 | *Borrelia burgdorferi* (GenBank # CP152378.1) | 198 | 198 | 100% | 9e-47 | 100% |
| Nested PCR | *ospC* | L3g | 1 | *Borrelia burgdorferi* (GenBank # U01894.1) | 418 | 418 | 100% | 2e-112 | 100% |
| | | | 2 | *Borrelia burgdorferi* (GenBank # JQ308224.1) | 418 | 418 | 100% | 2e-112 | 100% |

| | | | | | | | |
|---|---|---|---|---|---|---|---|
| | 3 | *Borrelia burgdorferi* (GenBank # AF411451.1) | 418 | 418 | 100% | 2e-112 | 100% |
| L3p | 1 | *Borrelia burgdorferi* (GenBank # U01894.1) | 412 | 412 | 100% | 7e-111 | 100% |
| | 2 | *Borrelia burgdorferi* (GenBank # JQ308224.1) | 412 | 412 | 100% | 7e-111 | 100% |
| | 3 | *Borrelia burgdorferi* (GenBank # AF411451.1) | 412 | 412 | 100% | 7e-111 | 100% |
| L35g | 1 | *Borrelia burgdorferi* (GenBank # U01894.1) | 385 | 385 | 100% | 1e-102 | 100% |
| | 2 | *Borrelia burgdorferi* (GenBank # JQ308224.1) | 385 | 385 | 100% | 1e-102 | 100% |
| | 3 | *Borrelia burgdorferi* (GenBank # AF411451.1) | 385 | 385 | 100% | 1e-102 | 100% |
| L70g | 1 | *Borrelia burgdorferi* (GenBank # U01894.1) | 381 | 381 | 100% | 2e-101 | 100% |
| | 2 | *Borrelia burgdorferi* (GenBank # JQ308224.1) | 381 | 381 | 100% | 2e-101 | 100% |
| | 3 | *Borrelia burgdorferi* (GenBank # AF411451.1) | 381 | 381 | 100% | 2e-101 | 100% |
| L72g | 1 | *Borrelia burgdorferi* (GenBank # U01894.1) | 427 | 427 | 100% | 3e-115 | 100% |
| | 2 | *Borrelia burgdorferi* (GenBank # JQ308224.1) | 427 | 427 | 100% | 3e-115 | 100% |
| | 3 | *Borrelia burgdorferi* (GenBank # AF411451.1) | 427 | 427 | 100% | 3e-115 | 100% |
| L73g | 1 | *Borrelia burgdorferi* (GenBank # U01894.1) | 431 | 431 | 100% | 2e-116 | 100% |
| | 2 | *Borrelia burgdorferi* (GenBank # JQ308224.1) | 431 | 431 | 100% | 2e-116 | 100% |
| | 3 | *Borrelia burgdorferi* (GenBank # AF411451.1) | 431 | 431 | 100% | 2e-116 | 100% |
| L99g | 1 | *Borrelia burgdorferi* (GenBank # U01894.1) | 427 | 427 | 100% | 3e-115 | 100% |
| | 2 | *Borrelia burgdorferi* (GenBank # JQ308224.1) | 427 | 427 | 100% | 3e-115 | 100% |
| | 3 | *Borrelia burgdorferi* (GenBank # AF411451.1) | 427 | 427 | 100% | 3e-115 | 100% |

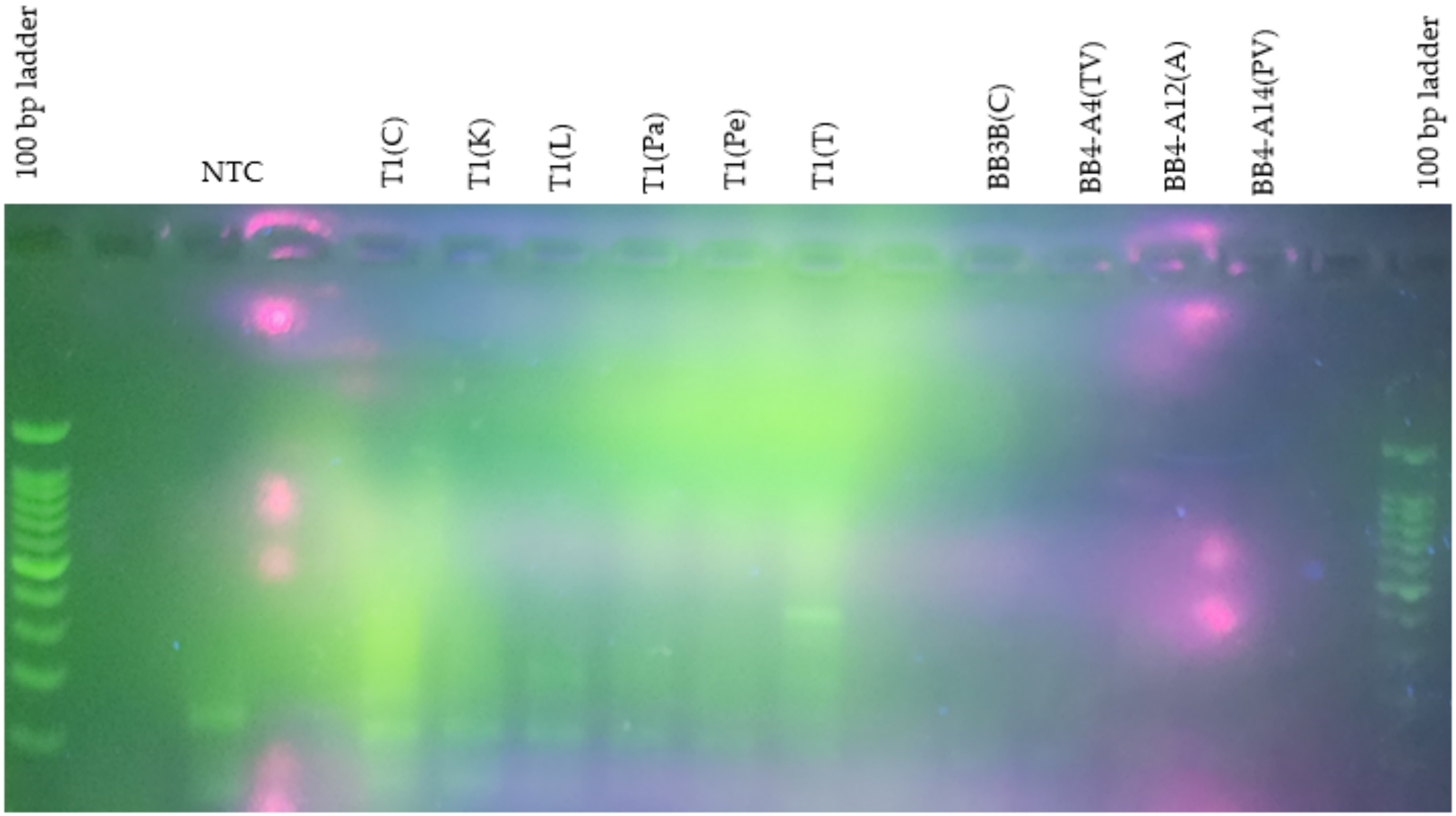


**Figure S1**. Gel electrophoresis of semi-nested amplicons from human tissues samples. Samples T1 and BB3 and BB4 are donor codes. The letters in parentheses indicate the tissue tested. "C" refers to a cortical sample, "K" refers to a kidney sample, "L" refers to a liver sample, "Pa" refers to a pancreatic tissue sample, "Pe" refers to a sample of pericardium, "T" refers to a tooth sample, "TV" refers to sample of the tricuspid valve, "A" refers to a sample of aorta and "PV" refers to a sample of the pulmonary vein. Expected amplicon size of the semi-nested ospA amplicon is 178 bp. NTC is a no template control.
